# Computer Vision Method for Automatic Detection of Microstructure Defects of Concrete

**DOI:** 10.3390/s24134373

**Published:** 2024-07-05

**Authors:** Alexey N. Beskopylny, Sergey A. Stel’makh, Evgenii M. Shcherban’, Irina Razveeva, Alexey Kozhakin, Besarion Meskhi, Andrei Chernil’nik, Diana Elshaeva, Oksana Ananova, Mikhail Girya, Timur Nurkhabinov, Nikita Beskopylny

**Affiliations:** 1 Department of Transport Systems, Faculty of Roads and Transport Systems, Don State Technical University, 344003 Rostov-on-Don, Russia; 2 Department of Unique Buildings and Constructions Engineering, Don State Technical University, 344003 Rostov-on-Don, Russia; sergej.stelmax@mail.ru (S.A.S.); razveevai@mail.ru (I.R.); alexeykozhakin@gmail.com (A.K.); chernila_a@mail.ru (A.C.); diana.elshaeva@yandex.ru (D.E.); giramisa6@gmail.com (M.G.); 3 Department of Engineering Geometry and Computer Graphics, Don State Technical University, 344003 Rostov-on-Don, Russia; au-geen@mail.ru; 4 OOO VDK, SKOLKOVO, Bolshoi Boulevard, 42, 121205 Moscow, Russia; 5 Department of Life Safety and Environmental Protection, Faculty of Life Safety and Environmental Engineering, Don State Technical University, 344003 Rostov-on-Don, Russia; spu-02@donstu.ru; 6 Department of Marketing and Engineering Economics, Faculty of Innovative Business and Management, Don State Technical University, 344003 Rostov-on-Don, Russia; o_ananova@mail.ru; 7 Department of Mathematical Theory of Intelligent Systems, Faculty of Mechanics and Mathematics, Lomonosov Moscow State University, Leninskiye Gory, 1, 119991 Moscow, Russia; timur.nurkhabinov@math.msu.ru; 8 Department of Hardware and Software Engineering, Faculty of IT-Systems and Technology, Don State Technical University, 344003 Rostov-on-Don, Russia; beskna@yandex.ru

**Keywords:** concrete, computer vision, convolutional neural networks, U-Net, LinkNet, PSPNet

## Abstract

The search for structural and microstructural defects using simple human vision is associated with significant errors in determining voids, large pores, and violations of the integrity and compactness of particle packing in the micro- and macrostructure of concrete. Computer vision methods, in particular convolutional neural networks, have proven to be reliable tools for the automatic detection of defects during visual inspection of building structures. The study’s objective is to create and compare computer vision algorithms that use convolutional neural networks to identify and analyze damaged sections in concrete samples from different structures. Networks of the following architectures were selected for operation: U-Net, LinkNet, and PSPNet. The analyzed images are photos of concrete samples obtained by laboratory tests to assess the quality in terms of the defection of the integrity and compactness of the structure. During the implementation process, changes in quality metrics such as macro-averaged precision, recall, and F1-score, as well as IoU (Jaccard coefficient) and accuracy, were monitored. The best metrics were demonstrated by the U-Net model, supplemented by the cellular automaton algorithm: precision = 0.91, recall = 0.90, F1 = 0.91, IoU = 0.84, and accuracy = 0.90. The developed segmentation algorithms are universal and show a high quality in highlighting areas of interest under any shooting conditions and different volumes of defective zones, regardless of their localization. The automatization of the process of calculating the damage area and a recommendation in the “critical/uncritical” format can be used to assess the condition of concrete of various types of structures, adjust the formulation, and change the technological parameters of production.

## 1. Introduction

### 1.1. Background

Currently, the search for innovative ways to recognize cracks and failure areas in the structure of cement concretes at the micro- and macrolevels is widely considered in scientific research and engineering practice. In building materials science, a fundamental relationship between the microstructure and properties of cement concretes was proven and confirmed by numerous studies. The structure, in turn, is considered as a set of properties and characteristics of the material in each unit of the volume of this material and at the micro- and macrolevels, forming the final quality of the cement composite [1].

Structural defect detection is a difficult process, depending on many conditions and to a large extent on the human factor in terms of the application of simple human vision [2,3,4,5]. Advertence is required during the determination of voids in the micro- and macrostructure of concrete, large pores, and violations of the integrity and compactness of the packaging of particles forming the structure of concrete. There are non-destructive methods of defect detection [6] such as FEM (finite element method) [7], ultrasonic [7,8,9], and electro-impedance spectroscopy [10] based on the use of various devices and sensors [11]. These methods have shown themselves to work well but are limited in applicability at the early stage of diagnostics when structural defects are microscopic and have not yet reached the surface.

Computer vision (CV) methods, in particular convolutional neural networks (CNN), have proven to be reliable instruments for the automatic detection of defects during visual inspection of building structures [12,13,14,15,16]. The U-Net and PSPNet CNN architectures demonstrate high accuracy in solving real-world application tasks in the presence of a small amount of data. In the study [17], the trained CNN AlexNet is integrated into a mobile smartphone application to make the process of examining concrete structures more accessible in practice. The results showed that the average accuracy on 205 images is 99.09%. It should be noted that the images in the test sample are different in their visual component, while the algorithm copes with each of them, spending ~60 s on processing and analysis.

Several works reflect the application of computer vision methods at the level of micro-cracks [18]. In [19], to identify (with an accuracy of 0.992), quantify, and visualize micro-cracks in high-performance fiber-reinforced cement composites (HPFRCC), several deep learning models and computer vision methods are combined into a hierarchical architecture. The authors note the prospect of using this method for other materials in which complex cracks are observed. In the study [20], when analyzing samples of hardened cement paste for the presence of cracks, indicating the beginning of destruction at the microlevel, the segmentation method using models based on the U-Net CNN is used. The accuracy of the models stated in this work was at least 60%, which meets the needs of the technologist in analyzing the structure of the composite and has practical application value.

CV methods can be part of an automatic segmentation system, complementing widely used methods for analyzing the microstructure of concrete [21,22,23,24,25,26,27,28]. In [17], scanning electron microscopy (SEM) was supplemented with a deep segmentation algorithm. The combination of these technologies has made it possible to achieve high accuracy, especially around the boundaries of areas of interest, which is a problem area in such tasks. In [19], X-ray computed tomography (CT) was supplemented by the stage of processing CT images using the deep convolutional neural network. In addition, CV methods allow for the detection of defects on the surface of materials [29,30,31], measuring geometric parameters of defects inside materials (in the structure) [32,33,34], producing a picture of clear boundaries of defects [35,36,37,38,39], and classifying defects [40,41].

### 1.2. Rationale

The literature analysis reveals a necessity to enhance and add to tools that automatically detect faulty areas. Computer vision methods are considered by researchers as the way to improve systems for analyzing the structure of concrete. Existing methods, for all their advantages, have a limited range of applications. Therefore, to determine the size of voids, large pores, failures of the integrity, and compactness of particle packing in the micro- and macrostructure of concrete, other labor-intensive, and expensive methods are used. An intelligent approach allows us to automate the process as much as possible and connect it with BIM technologies, which are actively developing in construction. It should be noted that applying computer vision methods to micro- and macrophotographs of the structure of building materials has not been sufficiently studied in the literature. The scientific novelty of the research lies in the development of new computer vision algorithms based on convolutional neural networks to identify violations of the integrity and compactness of the concrete micro- and macrostructure and the influence of various formulation and technological factors on the formation of defects in the concrete structure.

Microscopic analysis, used in building materials science in building materials technology, has become a good solution for searching and detecting structural defects, namely pores and voids, reducing the compactness of structure particles, which is noticeable to the human eye in the form of a change in the color scheme in areas where these defects are present. If there are dark spots in the photographs of the concrete structure (areas with a darker shade, different from the bulk of the material), we can examine the presence of defects. There is a clear relationship expressed in the shade’s darkness of the defective area. The darker the area being considered, the stronger the localization of defects in that place becomes. Consequently, the density of packing particles weakens and lowers, leading to a decrease in the characteristics of concrete. This can cause a critical situation where the concrete becomes inoperable. Due to the difficulty and complexity of the human eye in searching and interpreting darkened areas, it seems advisable to use computer vision methods to search for such defects, and later to interpret damage by dark shades.

The method proposed in this study implies the implementation of algorithms based on convolutional neural networks of various architectures, which, after training on a representative sample of samples, can work fully as an identifier of defects in the material’s structure. After conducting an analysis at the intelligence level of the machine, it is supposed to issue the simplest conclusion on the suitability of the analyzed concrete sample for operation on the principle of “critical/uncritical”. This recommendation will simplify the process of composition development and assessment of violations of the integrity and compactness of the concrete structure. The objective of the study is to develop and compare computer vision algorithms based on convolutional neural networks for segmenting defective areas in concrete samples of various structures. The steps required to achieve the key objective are as follows:

- the formation of a database “Photographic images of the microstructure of concrete”, describing the quality of concrete samples during laboratory experiments;
- description and implementation of CNN models based on LinkNet, U-Net, and PSPNet architectures;
- optimization and testing of implemented models taking into account segmentation quality requirements;
- processing of the results using “cellular automata”;
- visual assessment of the results obtained and comparison with the assessment put forward by a technologist;
- development of recommendations on the use and scaling of the proposed algorithms;
- assessment of the prospects for the introduction of CV algorithms into practice in assessing the quality of finished samples, as well as in the process of developing formulations.

## 2. Materials and Methods

### 2.1. Materials

The manufacture of heavy concrete involves the following materials.

(1) Portland cement (PC) CEM I 42.5N (CEMROS, Stary Oskol, Russia), which has the following properties:
  - specific surface area—335 m^2^/kg;
  - fineness, passage through a sieve No 008—98.6%;
  - start of setting—190 min;
  - end of setting—280 min;
  - compressive strength—19.1 MPa (after 2 days) and 51.3 MPa (after 28 days);
(2) quartz sand (QS), which has the following properties:
  - fineness modulus—2.19;
  - bulk density—1351 kg/m^3^;
  - apparent density—2630 kg/m^3^;
  - the content of dust and clay particles—0.04%;
  - content of clay in lumps—0.01%;
(3) crushed sandstone (CrS) (RostMed, Kamensk, Russia) with the following properties:
  - bulk density—1402 kg/m^3^;
  - apparent density—2638 kg/m^3^;
  - resistance to fragmentation—11.8 wt%;
  - the content of lamellar and acicular grains—7.7 wt%;
(4) plasticizing additive (PA) MasterGlenium 115 (BASF Construction Systems, Moscow, Russia):
  - color—light yellow;
  - density—1064 kg/m^3^;
  - PH—5.04;
  - the added amount is 0.5% of the weight of Portland cement.

The proportions of the concrete mix per 1 m^3^ are as follows:

PC—340 kg/m^3^; water—190 L/m^3^; QS—690 kg/m^3^; CrS—1090 kg/m^3^; PA—3.5 kg/m^3^.

The concrete itself has the following characteristics:

(1) density—2300 ± 40 kg/m^3^;
(2) the draft of the cone is from 3 to 5 cm;
(3) compressive strength—47.1 ± 2.2 MPa;
(4) water absorption—6.74 ± 0.36%.

### 2.2. Methods

A database called “Photographic images of concrete microstructure” was formed as a result of laboratory tests to assess concrete quality based on particle packaging and structural integrity [42]. The following equipment was used for this purpose:

- concrete mixer BL-10 (ZZBO, Zlatoust, Russia);
- CSF vibration platform (IMash, Armavir, Russia);
- normal hardening chamber KNT-1 (RNPO Rusuchpribor, St. Petersburg, Russia);
- hydraulic press P-125 (PKC ZIM, Armavir, Russia);
- optical microscope MBS-10 (Izmeritelnaya Tekhnika, Moscow, Russia) with magnification up to 10 times.

**Figure 1:**
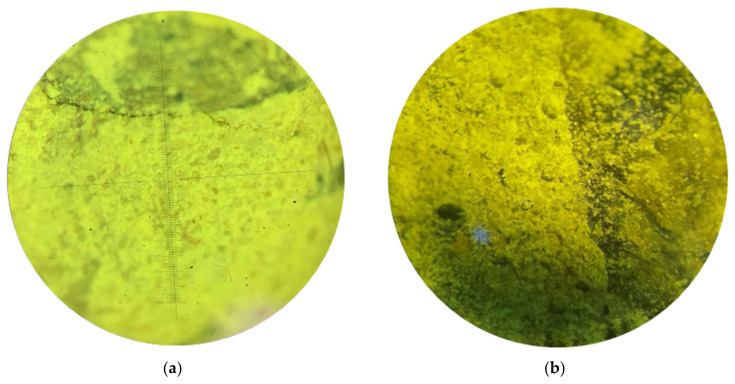
Photographs of concrete structure: (**a**) sample 1; (**b**) sample 2.

Figure 1a,b shows sample images.

It is worth noting the different degrees of illumination of the images and the excellent location of the defective areas. Failure of the structure of concrete samples is characterized by the following defects: voids in the concrete body (cavities), channels (cracks), and deep shells. These failures of the structure of the samples may be caused by poor-quality raw materials, under-compaction of concrete, or improper selection of the composition of concrete, that is, various prescription and/or technological aspects. The segmentation algorithm should be universal and show a high quality of highlighting areas of interest under any shooting conditions and different volumes of defective zones, regardless of their localization.

Convolutional neural networks act as the basis for CV algorithms, providing a high level of efficiency and accuracy in all areas of the construction industry, which has been proven in practice [43,44]. CNN of the following architecture is selected: LinkNet, U-Net, and PSPNet.

**Figure 2:**
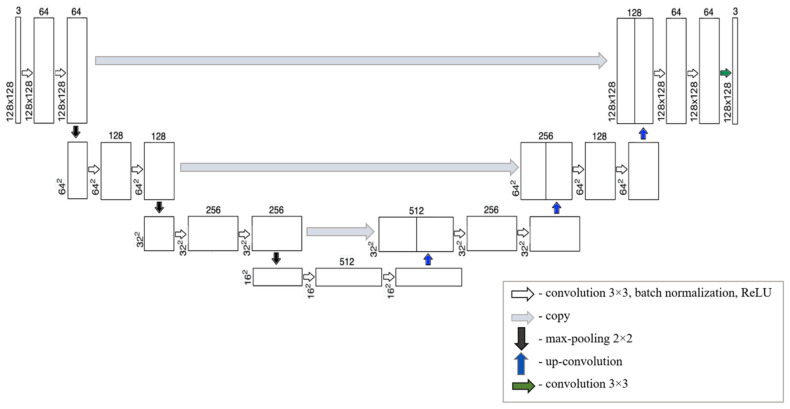
U-Net.

U-Net, created in 2015 [45], is a convolutional neural network architecture designed for image segmentation tasks. The U-Net architecture comprises two main parts: an encoder and a decoder. The encoder performs context capture and high-level object extraction from the input image, allowing the decoder to reconstruct the segmented output image using layers borrowed from the encoder block. Figure 2 depicts the structure of the U-Net network, comprising a narrowing path on the left and an expanding path on the right. Arrows denote different operations.

Because the stored low-level spatial information is used at the stage of increasing sampling, the U-Net CNN can segment small structures, such as pores and small particles well, which is necessary in this study.

**Figure 3:**
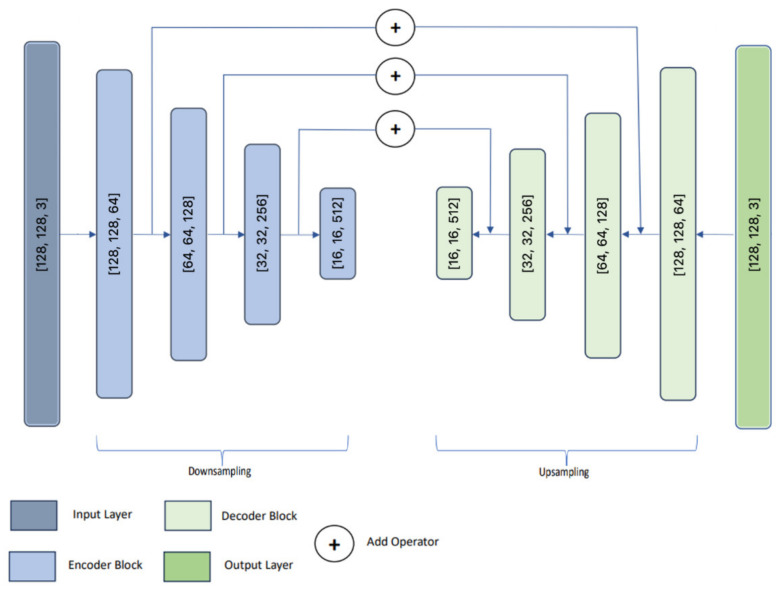
LinkNet.

The second architecture chosen for this study is LinkNet (Figure 3).

A feature of the architecture is that the output of each encoder level is transmitted to the input of the corresponding decoder [46,47]. Through this approach, our aim is to restore the spatial information that has been lost, allowing the decoder to benefit from it during sampling enhancement. This network becomes more efficient due to fewer parameters and is useful, including in real time.

**Figure 4:**
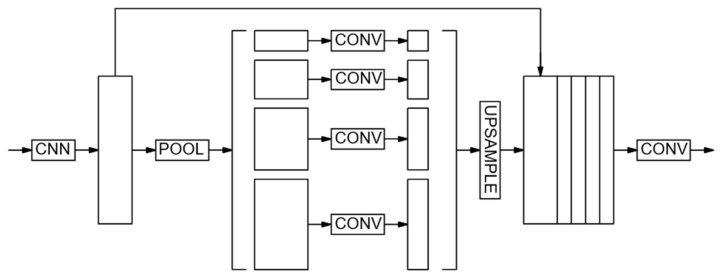
PSPnet-v1.

The next architecture used is PSPNet [48]. The model is optimized for deep image study, can segment objects of different scales, and is widely used as a basis for modified networks [49]. Two versions of the CNN are constructed in the study. In PSPNet-v1 (Figure 4), the image is transmitted to the input convolutional block to obtain a feature map. Next, the feature map is narrowed down to four different scales using pooling layers of different core sizes. Then convolutions are applied, after which all feature maps are expanded by up-sampling layers to the total size of the matrices and joined together. Finally, the output convolutional block is used to produce the final segmented image.

**Figure 5:**
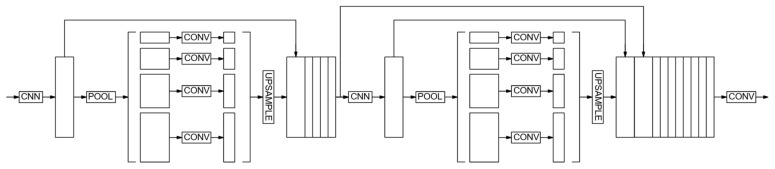
PSPNet-v2.

During the work, the PSPNet-v2 architecture based on PSPNet was also built (Figure 5). It consists of a PSPNet, the penultimate layer of which is remembered.

The output of the first PSPNet is transmitted to the second PSPNet. The stored layer is concatenated (connected) to the penultimate layer of the second PSPNet. The modified architecture showed a slight advantage in terms of metrics, which will be reflected in Section 3.

In total, 4 convolutional neural networks were used in this study to segment defective areas in concrete samples: LinkNet, U-Net, PSPNet-v1, and PSPNet-v2.

## 3. Results and Discussion

### 3.1. Model Training

The database “Photographic images of the microstructure of concrete” was used for training, validation, and testing of computer vision models. The dataset was augmented to include 500 images using the author’s code [50]. The augmentation process allows one to diversify the sample as much as possible, making it more resistant to noise and changes in shooting conditions. The resulting dataset was divided into 350/100/50 training, validation, and test samples, respectively.

**Figure 6:**
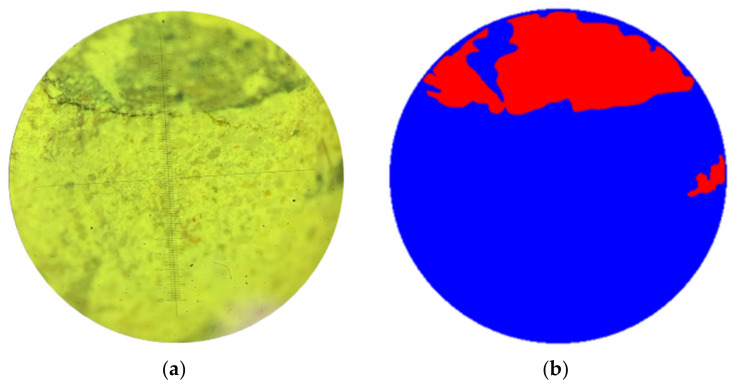
Photograph of concrete structure: (**a**) original image; (**b**) mask.

Before submitting data to the input of computer vision models, the technologist carried out the process of marking images, where the damaged area of interest was highlighted according to the expert. The resulting masks were saved in .png format and had the same size as the image they annotate. Figure 6 demonstrates an example of the original image and its mask, where a defective area in a heavy concrete sample (class 1) is indicated in red, an undamaged area (class 2) is blue, and the background (class 3) is white.

After the formation of image masks, the selected CNN models were trained. Training, optimization, and testing were carried out in the high-level Python 3.8 language using the TensorFlow v2.15.0 library. This library is a powerful, flexible, and effective tool for deploying algorithms based on convolutional neural networks, while it is possible to perform distributed calculations when training models.

Table 1 shows the parameters for training convolutional neural networks selected in this study.

For an objective comparison of models, parameters such as batchSize and number of epochs were set the same and equal to 50 and 200, respectively. The Jaccard loss function was used as a loss function for all models, which was minimized using the Adam stochastic optimization method [51]. Adam’s method, as a rule, converges faster to the optimal solution compared to other optimizers, such as SGD, AdaGrad, and RMSProp. In addition, this optimizer is less prone to fluctuations and more resistant to local minima, which makes it suitable for many tasks related to deep learning. That is why we used this optimizer in implementing this study. To adaptively adjust the learning rate during the training of models, the ReduceLROnPlateau method was used, according to which the learning rate automatically decreases when the model stops showing improvement according to a certain metric (in our case, Jaccard loss) (https://keras.io/api/callbacks/reduce_lr_on_plateau/, accessed on 12 May 2024).

During the implementation process, changes in the following quality metrics were monitored: the average values of the *Precision*, *Recall*, *F*1 metrics, as well as *IoU* and *Accuracy* [52,53]. The calculations of these metrics are shown in Formulas (1)–(5):

(1) $$Precision_{M}=\frac{\sum_{i=1}^{l}\frac{tp_{i}}{tp_{i}+fp_{i}}}{l}$$

(2) $$Recall_{M}=\frac{\sum_{i=1}^{l}\frac{tp_{i}}{tp_{i}+fn_{i}}}{l}$$

(3) $$F1score_{M}=\frac{\left(\beta^{2}+1\right)Precision_{M}Recall_{M}}{\beta^{2}Precision_{M}+Recall_{M}}$$

(4) $$IoU=\frac{\left|X\cap Y\right|}{\left|X\cup Y\right|}$$

(5) $$Accuracy=\frac{M}{N}$$

where *tp_i_* is the number of pixels of the image correctly assigned by the model to class *i*, where *i* = 1, 2, and 3;

*fp_i_* is the number of pixels of the image assigned by the model to class *i*, but which does not actually belong to this class, *i* = 1, 2, 3;

*fn_i_* is the number of pixels in the image that are not assigned to class *i* by the model, but which actually belong to this class, *i* = 1, 2, 3;

*l* is the number of classes in the image (in our case *l* = 3);

β is a positive factor (in the case of F1 β = 1);

*X* is the set of pixels of the image defined by the markup as an area of a certain class;

*Y* is a set of image pixels belonging to an area of a certain class according to the conclusions of the developed model;

*M* is the number of pixels of the image correctly marked by the model;

*N* is the number of all pixels in the image.

**Figure 7:**
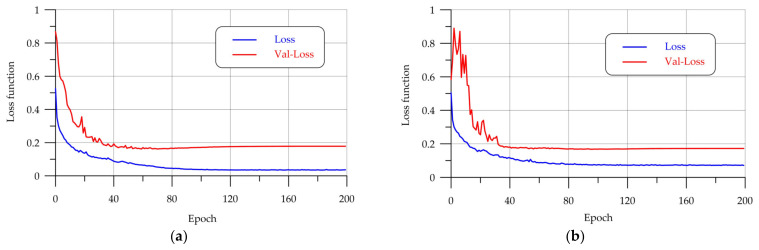

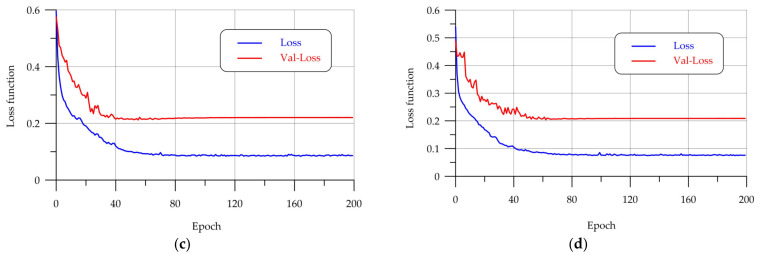
Training: (**a**) U-Net; (**b**) LinkNet; (**c**) PSPNet-v1; (**d**) PSPNet-v2.

Figure 7 shows the process of training models. Tracking error reduction in training convolutional neural networks is an important element in the model learning process. Changing the value of the loss function allows you to evaluate how well the model is trained. The decrease in the error value reflects an improvement in the model’s accuracy.

The graphs on the OY axis show the error values in the validation and training samples (yellow and blue lines, respectively), and on the OX axis the number of epochs. These graphs visualize the reduction of errors in the learning process. Tracking the error drop helps to determine the moment when the model is sufficiently trained. The optimal number of training epochs for the implemented models is about 100 epochs, after which the graph of the loss function reaches a plateau.

**Figure 8:**
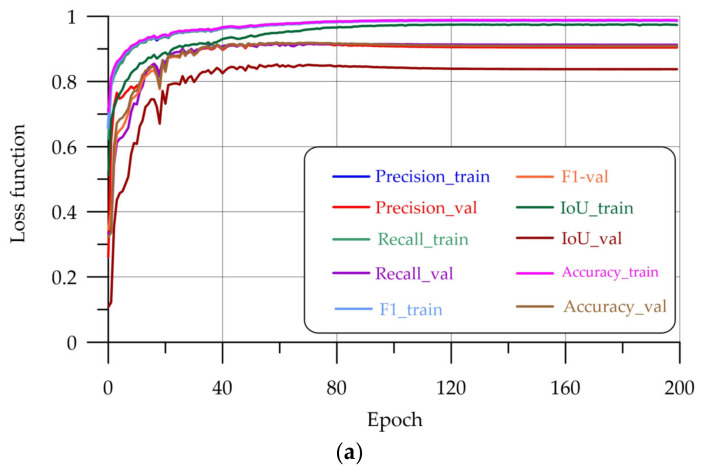

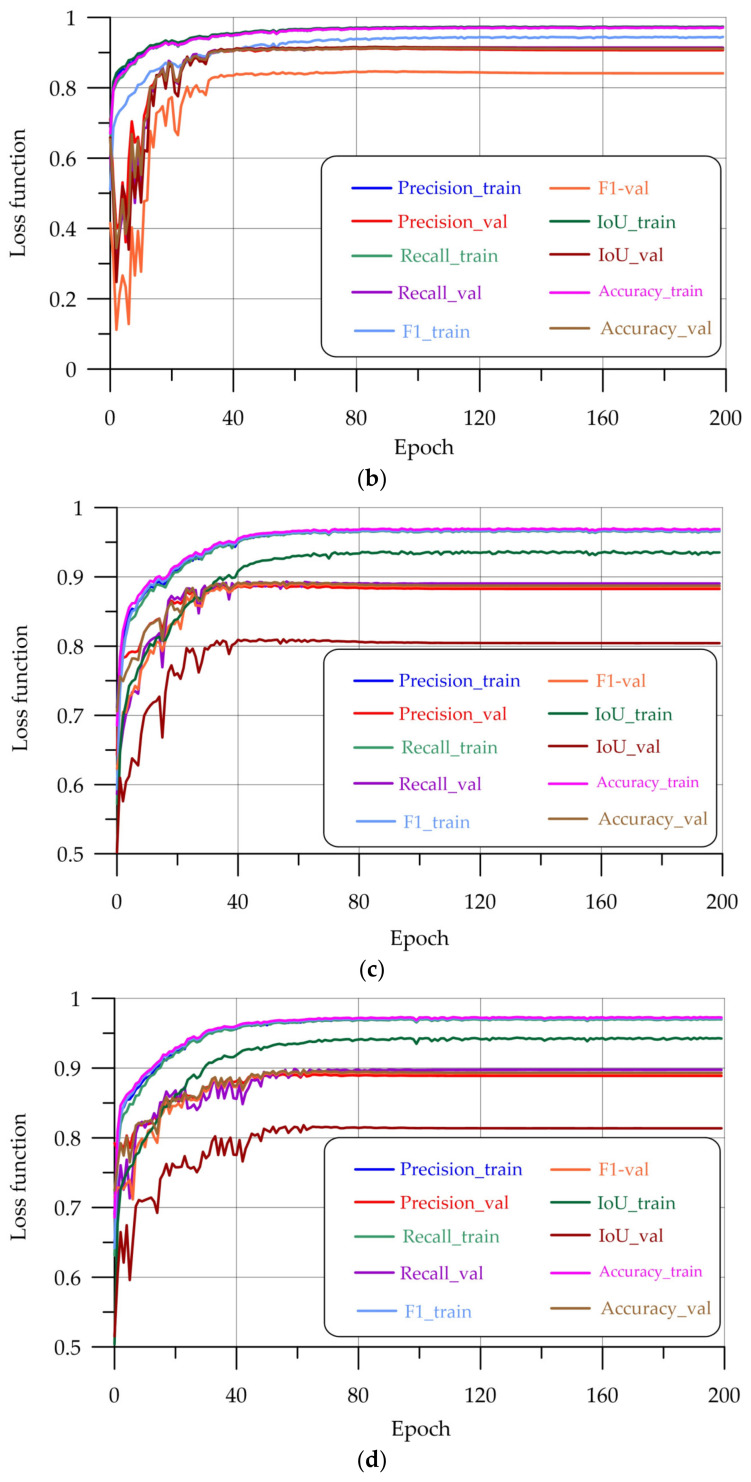
Metric graphics: (**a**) U-Net; (**b**) LinkNet; (**c**) PSPNet-v1; (**d**) PSPNet-v2.

Figure 8 shows graphs of changes in segmentation quality metrics during model training. The values for the precision, recall, F1, IoU, and accuracy metrics are reflected on both the training and validation samples calculated at each epoch.

### 3.2. Evaluation of Results

An analysis of the graphs shows that the metrics consistently rise as learning progresses. At around the 100th epoch, the metrics in the validation sample cease to increase. To improve the stability of the learning quality, the ReduceLROnPlateau method was used. If the loss function on the validation sample did not improve during a given number of patience steps, then the learning rate was multiplied by the “factor” factor from Table 1. The initial and maximum possible learning rate was also set—min_lr and max_lr, respectively. Due to the use of this method, the learning schedules are visually smooth as you learn.

**Figure 9:**
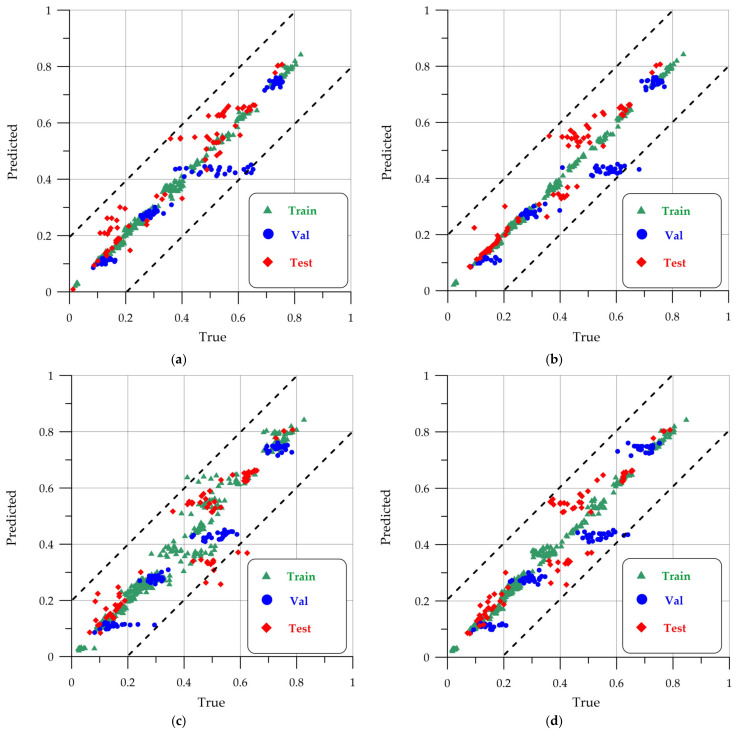
Dispersion graph for the “defect” class: (**a**) U-Net; (**b**) LinkNet; (**c**) PSPNet-v1; (**d**) PSPNet-v2.

Figure 9a–d show the dispersion graph for the training, validation, and test sample for Class 1—“defect”. For each image point, fractions of the area of the “defect” class are deposited on the axes: OX—true, OY—pred.

Table 2 shows the final quality metrics for the implemented models.

According to the table, the best results are demonstrated by the U-Net CNN. This is due to the fact that the architecture of this network contributes to better feature extraction and helps to cope with the problem of spatial information loss. This model also has the ability to capture textures in the image well. Slightly inferior to U-Net in terms of CNN metrics of the PSPNet-v2 architecture. The accuracy of the implemented models started from 0.89.

**Figure 10:**
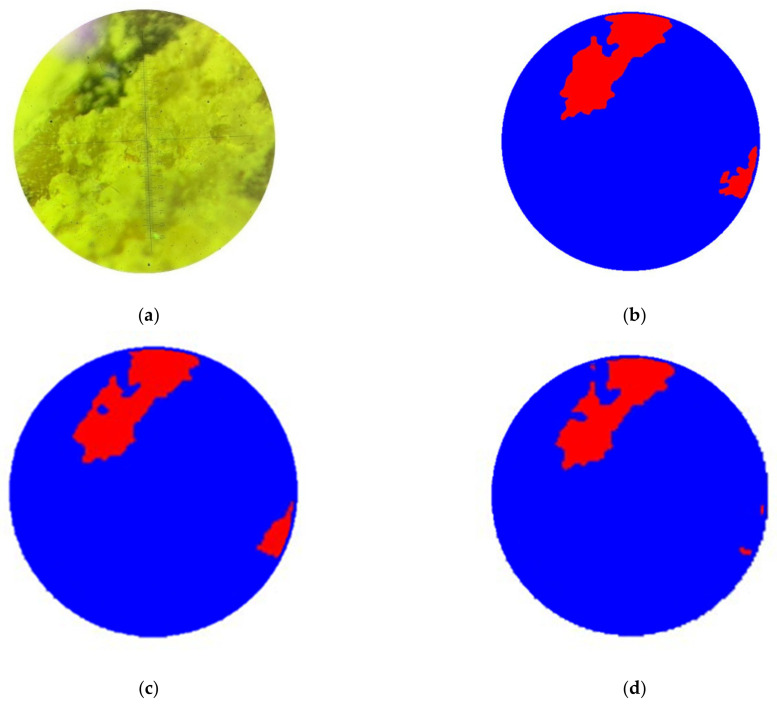

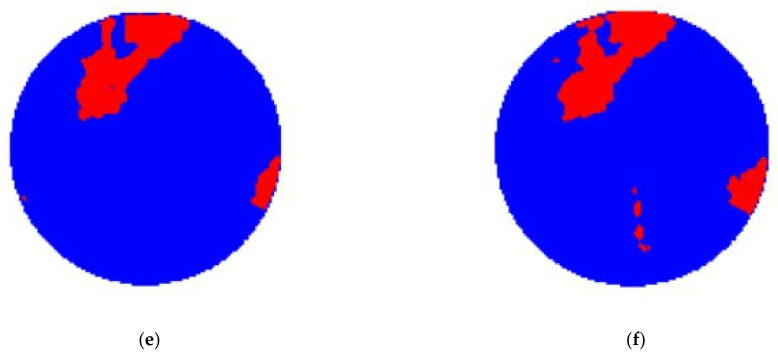
Segmentation result: (**a**) the original image; (**b**) the original mask; (**c**) segmentation by the U-Net model; (**d**) segmentation by the LinkNet model; (**e**) segmentation by the PSPNet-v1 model; (**f**) segmentation by the PSPNet-v2 model.

Figure 10 demonstrates the results of segmentation in the images of the test sample: the original image, its mask (created manually by a technologist), and the segmented image as a result of the work of each of the models.

It follows from the visual inspection that the U-Net CNN has captured the boundaries of the damaged area in the best way.

### 3.3. Post-Processing by Cellular Automaton

To obtain a smoother and clearer contour, the cellular automata algorithm was applied, which made it possible to remove noise and make the segmented area more complete.

Let the pixels of the area of interest have a value of 1, and the pixels of the background have a value of 0. Then we can introduce a cellular automaton (Z^2^, $E_{2}$, V, φ), in which Z^2^ is the set of all two-dimensional vectors with integer coordinates called cells, $E_{2}$ = {0, 1} is the set of cell states of the cellular automaton, V is an ordered set of nonzero pairwise distinct two-dimensional vectors with integer coordinates, called a neighborhood.

Let us write the transition function φ(x_0_) as follows:

$$\varphi \left(x_{0}\right)=\left\{\begin{array}{c} 1,\;if\;\left(\overset{8}{\underset{i=1}{⋁}}C_{i}\right)\vee \left(\overset{4}{\underset{j=1}{⋁}}C_{j}\right)=1\;\\ 0,\;if\overset{8}{\underset{l=1}{⋁}}C_{l}=1 \end{array}\right.$$

$$C_{i}=\overset{p}{\underset{i_{n}=1}{⋀}}x_{i_{n}}$$

$$C_{l}=\overset{s}{\underset{l_{n}=1}{⋀}}{\bar{x}}_{l_{n}}$$

p, s is the number of cells in the parts of the neighborhood.

The parts $\vee_{i},\vee_{l}$ of the neighborhood template have the following form:

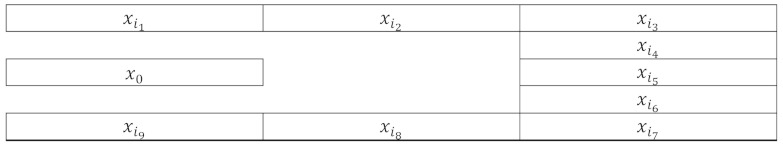

$$C_{j}=x_{j_{1}}\wedge \;x_{j_{2}}$$

The $\vee_{j}$ part of the neighborhood template has the following form:

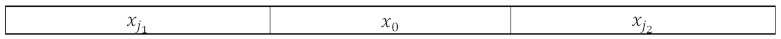


**Figure 11:**
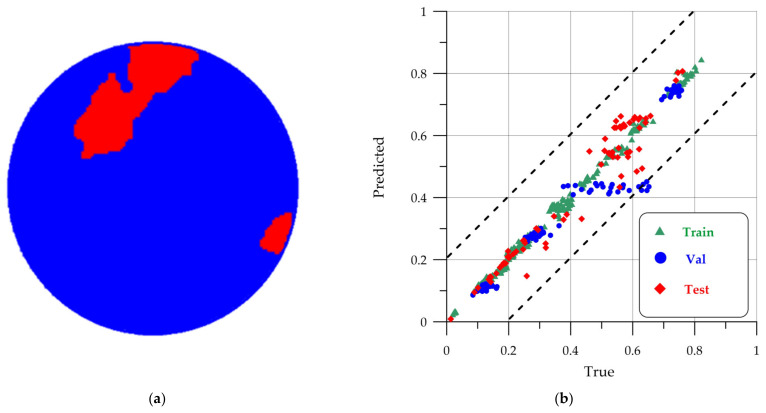
The result of using a cellular automaton: (**a**) an improved mask; (**b**) an area dispersion graph.

The result of the cellular automaton algorithm is shown in Figure 11.

As can be seen from Figure 11a, the contour has become solid without visible gaps in the central part of the defect. The test points on the scatter plot Figure 11b have become closer to the *x* = *y* line, which indicates an improvement in segmentation. The final metrics after using the cellular automaton for the test sample for the U-Net model are: precision = 0.91, recall = 0.90, F1 = 0.91, IoU = 0.84, accuracy = 0.90.

### 3.4. Discussion

After the analysis by the computer vision algorithm, it is supposed to issue the simplest conclusion on the degree of suitability of the analyzed concrete sample for operation according to the principle of “critical/uncritical”. If the “defect” class occupies more than 20% of the area of the concrete sample, then a “critical” recommendation is issued. This recommendation indicates a violation of the integrity and compactness of the packaging of particles forming the structure of concrete, which means that an adjustment of the formulation or parameters of the concrete production technology is necessary.

Analysis of the research results and comparison of the developed algorithms with existing methods for assessing the quality of concrete showed that methods based on visual and instrumental assessments have a significant influence on the subjective opinion of the technologist. While algorithms based on convolutional neural networks are objective and oriented when making a decision on a given threshold of the percentage of the defective area.

The computer vision models considered in this study are not inferior in terms of quality metrics to the models proposed by researchers in [17,19,20], which makes it possible to talk about the competitiveness of the development. As in other works, this study notes the high speed of the algorithms, which allows you to analyze large amounts of data in short periods of time. In our case, the processing speed of a single image by a trained model is ~0.3 s. Damage classifiers using VGG19 and ResNet were used in [54]. The combined results showed a high accuracy of 86.7%. In our study, the best model achieved 90% accuracy. The quality of the model proposed in this study is also not inferior to the dynamic graph convolutional neural network model considered in [55], where the accuracy of detecting cracks and chips is 55.20% and 89.77%.

The algorithms developed in this study can be used in concretes of various structures—both conventional and with variatropic properties with different cross-section thicknesses of products and structures [56,57,58,59,60].

When implementing the developed intelligent algorithms, it is worth following the following recommendations:

- It is necessary to ensure transparency and a clear understanding of the results of the algorithms with the justification of the limits of acceptable errors to ensure the required level of strength. When issuing an opinion on the degree of suitability of the analyzed concrete sample for operation on the principle of “critical/uncritical”, it is necessary to be guided by current building codes and regulations. Users of the software product should have instructions on how to use smart algorithms and interpret segmentation results;
- When changing or supplementing the properties of materials affecting their structure, which can be detected by computer vision methods, it is advisable to use data drift technology, concept drift, and domain adaptation, which will allow taking into account new factors without completely retraining previously created models.

Practitioners can now use this approach to enhance their ability to detect different types of defects by taking the best model code as a basis. It can be built into a production line for real-time monitoring, or used locally on a computer for high-quality formulation development.

## 4. Conclusions

This article considers the methodology of creating CV algorithms based on convolutional neural networks of U-Net, LinkNet, and PSPNet architectures that allow for the segmentation of defective areas in concrete samples. The algorithm provides a recommendation on the strength characteristics of the analyzed sample. A proprietary empirical base is proposed for the study, which comprises photographs of the microstructure of heavy concrete samples formed in laboratory conditions during the assessment of the quality of integrity and compactness of the packaging of particles forming the composite structure. The results of the study led to the following conclusions.

(1) Three models of convolutional neural networks are implemented, one of which was modified by the authors.
(2) Training was carried out on our own dataset selected in laboratory conditions. The dataset has been enlarged using the author’s augmentation algorithm.
(3) The proposed machine vision algorithms have shown high accuracy (accuracy from 0.89) in detecting the area of interest.
(4) Evaluation of the quality of the results of the models suggests the following: the considered algorithms based on convolutional neural networks are, on average, able to detect at least 89% of all defects in photographs of concrete samples.
(5) A cellular automaton algorithm was proposed to post-process the segmentation results of the best model. The application of the cellular automaton algorithm made it possible to remove noise and make the segmented area more integral. The best metrics were demonstrated by the U-Net model, supplemented by this algorithm: precision = 0.91, recall = 0.90, F1 = 0.91, IoU = 0.84, accuracy = 0.90.
(6) The analysis of the segmentation results makes it possible to establish the relationship between the formulation, technological parameters, and the proportion of defects. The authorization of the process of calculating the damage area and a recommendation in the “critical/uncritical” format can be used to assess the condition of concrete of various types of structures, adjust the formulation, and change the technological parameters of production.

The study is planned to be continued in the following areas:

- expansion of the range of analyzed building materials by collecting new data during laboratory tests and in the course of fieldwork;
- application of convolutional neural networks of other architectures and/or modernization and hybridization of previously considered;
- combining the developed methods and traditional methods of defect detection into a single system, where one method will confirm or correct the conclusions of the other, guaranteeing the most reliable result;
- in-depth analysis of the strength properties of concrete from the parameters of defects in its microstructure (for example, from the color depth of the defective area);
- development of a user interface as a web platform for the convenience of interested parties’ access to this development. The user interface will allow you to apply the developed algorithms both locally on a computer in laboratories and in the field, where internet access is not always available. The web platform will allow you to access the algorithms from anywhere. This approach will satisfy all possible requests for this development.

## Figures and Tables

**Table 1:** Parameters for CNN.

№	Parameter	Parameter Description	U-Net	LinkNet	PSPNet-v1	PSPNet-v2
1	BatchSize	Size of training batch	50	50	50	50
2	Number of epochs	Number of epochs	200	200	200	200
3	max_lr	Maximum learning rate	0.0005	0.0005	0.0005	0.001
4	min_lr	Minimum learning rate	1 × 10^−7^	1 × 10^−7^	1 × 10^−7^	1 × 10^−7^
5	factor	The coefficient by which the learning rate is multiplied	0.7	0.7	0.7	0.7
6	patience	The number of epochs at which the loss function on validation data does not improve	5	5	5	4
7	Solver	Optimizer	Adam	Adam	Adam	Adam
8	Loss function	Loss function	Jaccard loss	Jaccard loss	Jaccard loss	Jaccard loss

**Table 2:** Final metrics.

№	Model Name	Precision	Recall	F1	IoU	Accuracy
1	U-Net	0.90	0.91	0.91	0.84	0.90
2	LinkNet	0.89	0.89	0.89	0.81	0.90
3	PSPNet-v1	0.90	0.89	0.88	0.81	0.89
4	PSPNet-v2	0.90	0.90	0.89	0.82	0.90

## Data Availability

Data are contained within the article.
